# Identification of Mubritinib (TAK 165) as an inhibitor of KSHV driven primary effusion lymphoma via disruption of mitochondrial OXPHOS metabolism

**DOI:** 10.18632/oncotarget.27815

**Published:** 2020-11-17

**Authors:** Abram Calderon, Samantha S. Soldan, Alessandra De Leo, Zhong Deng, Drew M. Frase, Emma M. Anderson, Yue Zhang, Olga Vladimirova, Fang Lu, Jessica C. Leung, Maureen E. Murphy, Paul M. Lieberman

**Affiliations:** ^1^The Wistar Institute, Philadelphia, PA 19146, USA

**Keywords:** KSHV, LANA, high-throughput screen (HTS), primary effusion lymphoma, Mubritinib

## Abstract

KSHV-associated cancers have poor prognoses and lack therapeutics that selectively target viral gene functions. We developed a screening campaign to identify known drugs that could be repurposed for the treatment of KSHV-associated cancers. We focused on primary effusion lymphoma (PEL), which has particularly poor treatment outcomes. We developed a luciferase reporter assay to test the ability of drugs to inhibit DNA binding of the KSHV LANA DNA binding domain (DBD). In parallel, we screened drugs for selective inhibition of a KSHV^+^ PEL cells. While potent hits were identified in each assay, only one hit, Mubritinib, was found to score in both assays. Mubritinib caused PEL cells to undergo cell cycle arrest with accumulation of sub-G_1_ population and Annexin V. Mubritinib inhibited LANA binding to KSHV terminal repeat (TR) DNA in KSHV^+^ PEL cells, but did not lead to KSHV lytic cycle reactivation. Mubritinib was originally identified as a receptor tyrosine kinase (RTK) inhibitor selective for HER2/ErbB2. But recent studies have revealed that Mubritinib can also inhibit the electron transport chain (ETC) complex at nanomolar concentrations. We found that other related ETC complex inhibitors (Rotenone and Deguelin) exhibited PEL cell growth inhibition while RTK inhibitors failed. Seahorse analysis demonstrated that Mubritinib selectively inhibits the maximal oxygen consumption (OCR) in PEL cells and metabolomics revealed changes in ATP/ADP and ATP/AMP ratios. These findings indicate that PEL cells are selectively sensitive to ETC complex inhibitors and provide a rationale for repurposing Mubritinib for selective treatment of PEL.

## INTRODUCTION

Kaposi’s Sarcoma-associated Herpesvirus (KSHV)/Human Herpesvirus 8 (HHV8) is a human γ-herpesvirus that establishes latent infection in B-lymphocytes and is strongly associated with several human cancers [1–3]. KSHV DNA and latency gene products are consistently detected in all forms of Kaposi’s sarcoma (KS), most forms of primary effusion lymphoma (PEL) and multicentric Castleman’s disease (MCD), and a percentage of B-cell lymphoproliferative diseases [4–6]. Introduction of KSHV DNA into mice causes KS-like malignancies, demonstrating that KSHV genes associated with latent infection promote the development of KS-like tumors [7–10]. KSHV is therefore considered the causative agent of KS and essential for the formation of PEL and MCD. At present, there are no KSHV-specific therapeutics approved for treatment of KSHV-associated cancers.

KSHV cancers consistently express a limited number of viral proteins that are associated with latent infection. During latency the KSHV genome is maintained by the viral Latency-Associated Nuclear Antigen (LANA), encoded by ORF73 [11–13]. LANA is a multifunction protein that plays a role in viral and cellular gene regulation, DNA replication, chromosome organization, cell cycle progression, and cell survival [14–16]. LANA, like EBNA1 from the Epstein Barr virus (EBV), is a DNA binding protein that binds and maintains viral genomes during latent infection [17]. LANA can also repress viral lytic genes, like ORF50 (RTA), and autoregulate its own transcription to maintain a stable latent infection [17, 18]. LANA has been shown to activate transcription of cellular oncogenes, such as hTERT, and repress tumor suppressors, like Rb, to promote oncogenic transformation [19, 20]. Furthermore, LANA is consistently detected in all KSHV-associated tumors [21]. Therefore, LANA is an attractive target for the development of KSHV-specific cancer therapeutics.

The mechanisms through which LANA maintains KSHV episomes during latent infection is known in some detail [14, 16]. LANA binds directly to a ~20 bp GC-rich sequence within the KSHV terminal repeats (TRs) [11–13, 22–24]. A minimal region of ~60 bp containing two LANA binding sites is sufficient to support transient DNA replication of plasmids [22]. Long-term episome maintenance requires an additional sequence from the TR and the presence of at least two TRs [24]. The C-terminal domain of LANA possesses sequence-specific DNA binding activity to the 18 bp element in the KSHV minimal replicator [22]. The X-ray structure of the DNA binding domain has been solved alone [25, 26] and in complex with bound TR DNA [27]. The LANA amino terminal domain interacts with histone H2A/H2B to tether itself to the metaphase chromosomes, which is required for episome maintenance [28]. Small molecules that bind to LANA have been explored [29] and identified [30–32]. But, to date, none of these have yet demonstrated efficacy *in vivo*.

**Figure 1:**
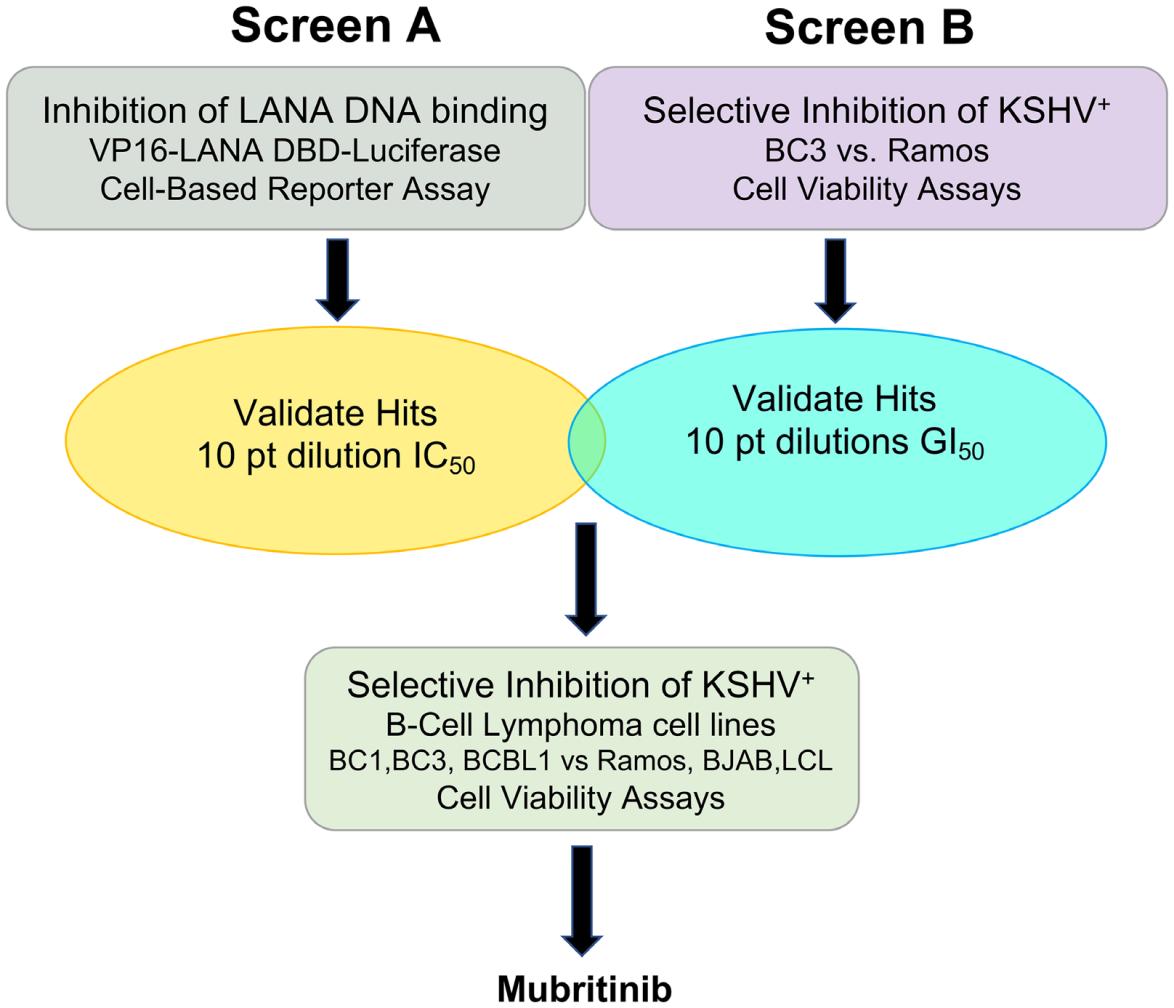
Drug screening strategy. Two primary screens one testing for inhibition of LANA DNA binding (Screen A) and another testing for selective cell growth inhibition of KSHV^+^ B-cell lymphoma (Screen B), were performed in parallel to identify LANA-specific inhibitors of KSHV positive tumors. Then, hits from these two screens were titrated against a panel of 6 B-cell lines. Mubritinib was identified as the best drug for further testing in KSHV^+^ PEL cells.

In an effort to find more efficacious treatments for KSHV-associated diseases, we have initiated a cell-based small-molecule screening campaign to identify inhibitors of LANA DNA binding that also inhibit KSHV positive cell growth. While LANA remains an attractive target for inhibition of KSHV-associated cancers, loss of LANA may not be sufficient to eradicate KSHV cancer cell growth. Genetic disruption of KSHV LANA leads to loss of viral episomes [33, 34], but it is not yet clear if this loss leads to an inhibition of cancer cell growth or tumorigenesis. Therefore, we set out to identify small molecule inhibitors of LANA DNA-binding that could also be readily tested for their ability to inhibit KSHV positive cell growth and tumorigenicity (Figure 1). To accomplish this, we screened a library of known drugs that could be repurposed for the treatment of patients with KSHV-associated cancers and related diseases.

## RESULTS

### Development of a cell-based assay for inhibition of LANA DNA binding

**Figure 2:**
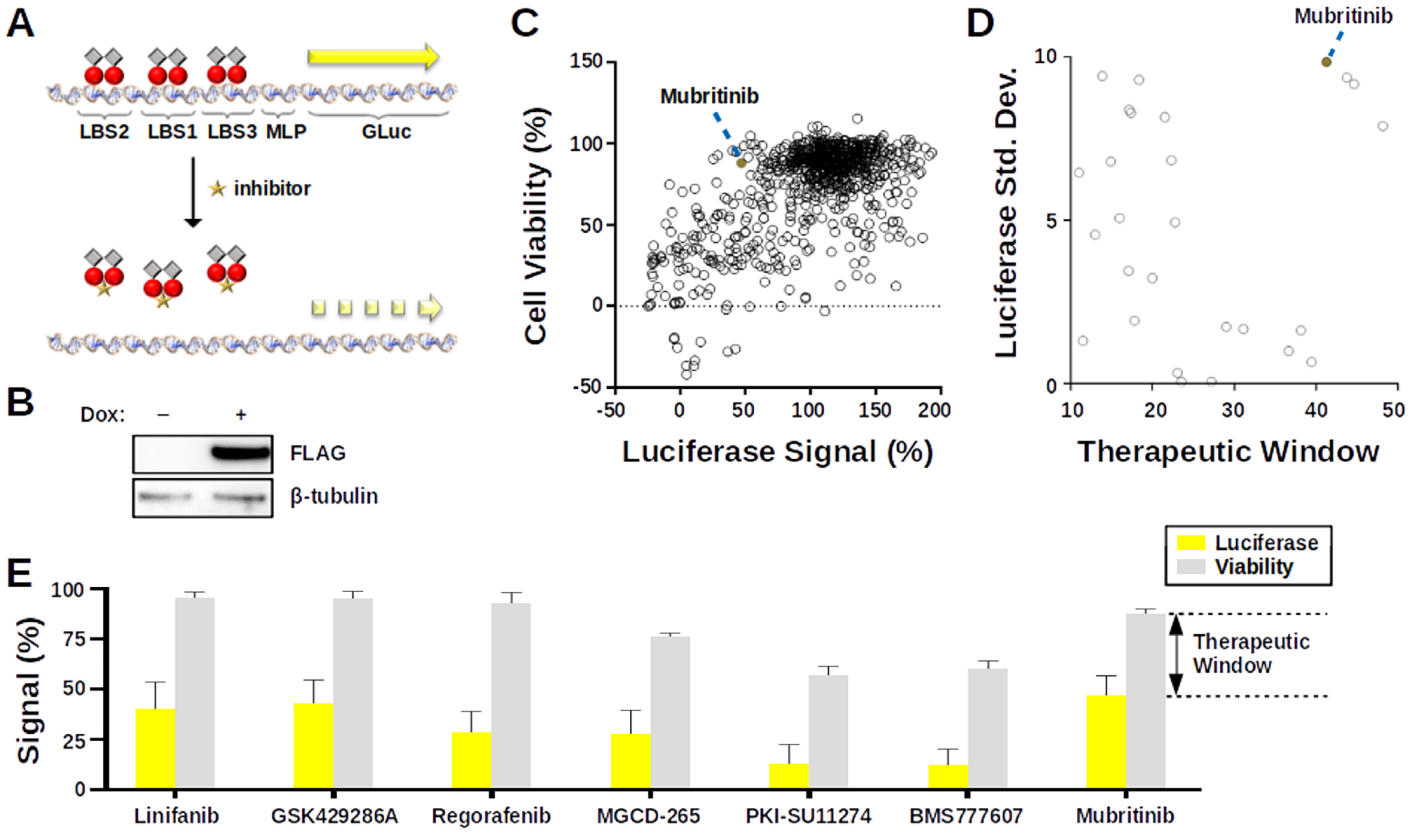
Cell-based screen for inhibitors of LANA DNA-binding. (**A**) Cartoon illustrating key features of the *Gaussia* luciferase assay. The reporter plasmid has three LANA Binding Sites (LBSs) adjacent to a Major Late Promoter (MLP) for the *Gaussia* luciferase (GLuc) gene. A fusion protein containing the Vp16 activation domain (gray diamond) and the LANA DNA-binding domain (DBD) (red circle) can bind to the LANA binding sites and enhance expression of GLuc (yellow arrow). However, in the presence of an inhibitor (gold star), LANA DNA-binding is reduced, causing a reduction in GLuc expression (dashed arrow). (**B**) Western blot demonstrating that expression of the FLAG-tagged Vp16-LANA DBD fusion protein is induced by doxycycline. Tubulin was used as a loading control. (**C**) Scatter plot showing the results of the luciferase screen. A large number of drugs (open circles) cluster near the upper right region of the graph, indicating that they had little effect on either the luciferase signal or the cell viability. Mubritinib is highlighted as a filled red circle. (**D**) Scatter plot focusing on drugs from the luciferase screen that have high therapeutic windows and highly reproducible luciferase signals. Mubritinib is highlighted as a filled red circle. (**E**) Graph summarizing data for top hit compounds from the luciferase screen as percentage of signal with DMSO control for Luciferase (yellow) or Resazurin viability assay (grey) relative to DMSO controls. The therapeutic index is calculated as the difference between the Resazurin and Luciferase signals.

We first developed a cell-based luciferase assay that would allow us to screen small molecules for their ability to inhibit LANA DNA binding (Figure 2A). To this end, we used the RMCE-HILO platform (41) to generate a stable HEK293T cell line that expresses a FLAG-tagged fusion protein containing the activation domain of the herpes simplex virus transcription factor VP16 and the LANA DNA-binding domain (DBD) upon induction with doxycycline (Figure 2B). We also generated a *Gaussia* luciferase reporter plasmid containing the three known LANA binding sites (LBS2, LBS1, and LBS3) from the KSHV TR region. In the presence of the LBS reporter plasmid, the fusion protein can bind to the LANA binding sites and transactivate expression of *Gaussia* luciferase. The extent of activation was sufficiently robust (~14-fold) for high-throughput screening. However, if an appropriate inhibitor is added to the media, the binding of the fusion protein will be reduced, causing a reduction in *Gaussia* luciferase expression.

### Identification of drugs that inhibit LANA DNA binding

The luciferase assay was used to screen a small library (~1,000 known drugs) from SelleckChem at a final concentration of 12.5 μM. Although a large number of these drugs had little effect on either cell viability or luciferase signal (Figure 2C, upper right cluster), there were also many drugs for which efficacy was more difficult to interpret. Therefore, we selected hits based on the inhibition of the luciferase signal relative to cell viability. This “therapeutic window” was calculated as the percent cell viability relative to DMSO control minus the percent luciferase signal relative to DMSO control (Figure 2D and 2E). Among the top hits, were several receptor tyrosine kinase (RTK) inhibitors, including Linifanib, Regorafenib, MGCD-265, BMS777606, and Mubritinib, and the ROCK-inhibitor GSK429286A (Figure 2E). We concluded that these drugs inhibit LANA DNA binding at micromolar concentrations in living cells.

### Screening for drugs that inhibit KSHV^+^ cell growth

**Figure 3:**
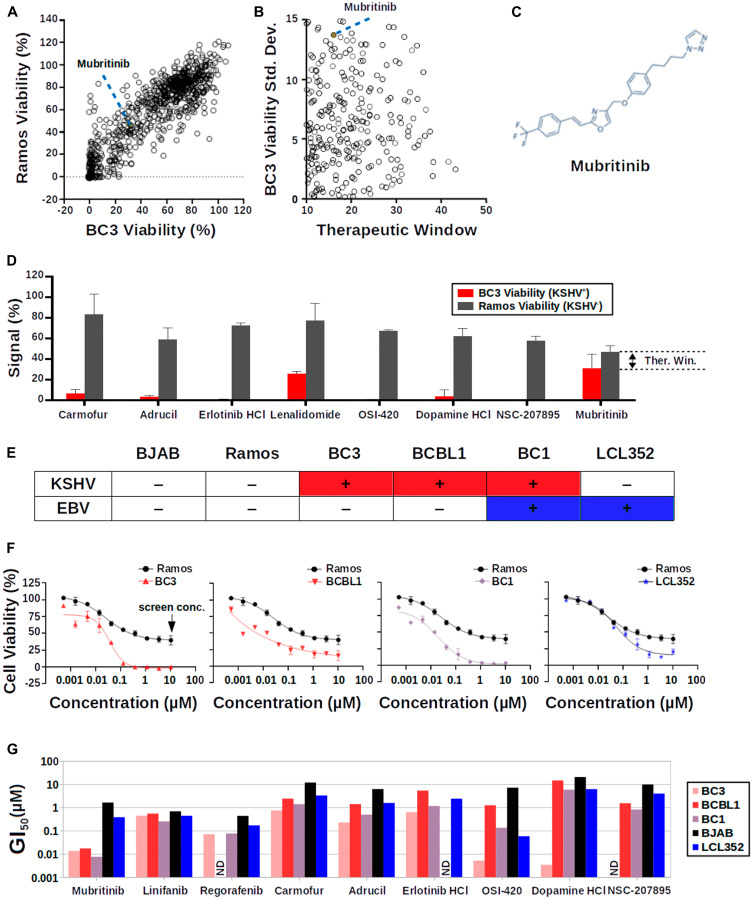
Cell-based screen for selective inhibition of PEL cell growth. (**A**) Scatter plot of the results of the cell growth inhibition screen. A large number of drugs (open circles) cluster along a diagonal line, indicating that they show similar effects on the viability of both Ramos (KSHV^-^) and BC3 (KSHV^+^) cells. Mubritinib is highlighted as a filled red circle. (**B**) Scatter plot focusing on drugs from the cell growth inhibition screens that have high therapeutic windows and highly reproducible BC3 viability signals. Mubritinib is highlighted as a filled red circle. (**C**) Chemical structure of Mubritinib. (**D**) Graph summarizing data for hit compounds from the cell growth inhibition screen. (**E**) Table summarizing the KSHV and EBV infection status of the B-cell lines used in this paper. (**F**) Titration of Mubritinib against various B-cell lines. Ramos (KSHV^-^, EBV^-^) data are shown for reference in each graph. KSHV^+^ cell lines (BC3, BCBL1, and BC1) respond more strongly to Mubritinib at nanomolar concentrations than the EBV^+^ (LCL352) cell line. (**G**) Graph summarizing the GI_50_ values for hits from the SelleckChem Library.

In parallel, we also screened the same SelleckChem library at a final concentration of 10 μM against two B-cell lines, a KSHV^-^ cell line (Ramos) and a KSHV^+^ PEL cell line (BC3) (Figure 3). As expected, most of the drugs showed similar effects on the viability of both of these cell lines (Figure 3A). Nevertheless, there were a small number of drugs that selectively reduced the viability of the KSHV^+^ BC3 cells relative to Ramos cells (Figure 3B). As with the luciferase assay, we chose hits based on a “therapeutic window”, which is simply the Ramos (KSHV^-^) percent cell viability minus the BC3 (KSHV^+^) percent cell viability, and the reproducibility of the BC3 viability signal (from data acquired on two separate days) (Figure 3C). Using this approach, we identified several drugs that selectively inhibited BC3 cell growth relative to Ramos cells (Figure 3C and 3D). Mubritinib was the only drug that appeared as a hit in both of the screens, even though its therapeutic window was relatively small.

### Mubritinib selectively inhibits PEL cell viability at nanomolar concentrations

Hits from both the luciferase and B-cell viability screens were assayed against a panel of six B-cell lines (Figure 3E) at ten different concentrations (Figure 3F). These data were used to determine growth inhibition index (GI_50_) values for each of these drugs (Figure 3G, Supplementary Table 1). Interestingly, only Mubritinib was found to reproducibly and selectively reduce the viability of KSHV^+^ PEL cell lines (BC3, BCBL1, and BC1) relative to KSHV^-^ cell lines (BJAB, Ramos, and LCL352) at nanomolar concentrations (Figure 3G). In particular, the GI_50_ values for Mubritinib in BC3, BCBL1, and BC1 cells were 13.45, 17.1, and 7.5 nM, respectively, while the GI_50_ values for BJAB, Ramos, and LCL352 cells were of 1.6, 0.4, and 0.2 μM, respectively. The results for the LCL352 cells are notable since these cells are infected with EBV, a γ-herpesvirus that is closely related to KSHV.

### Comparison of Mubritinib with other published PEL inhibitors

**Figure 4:**
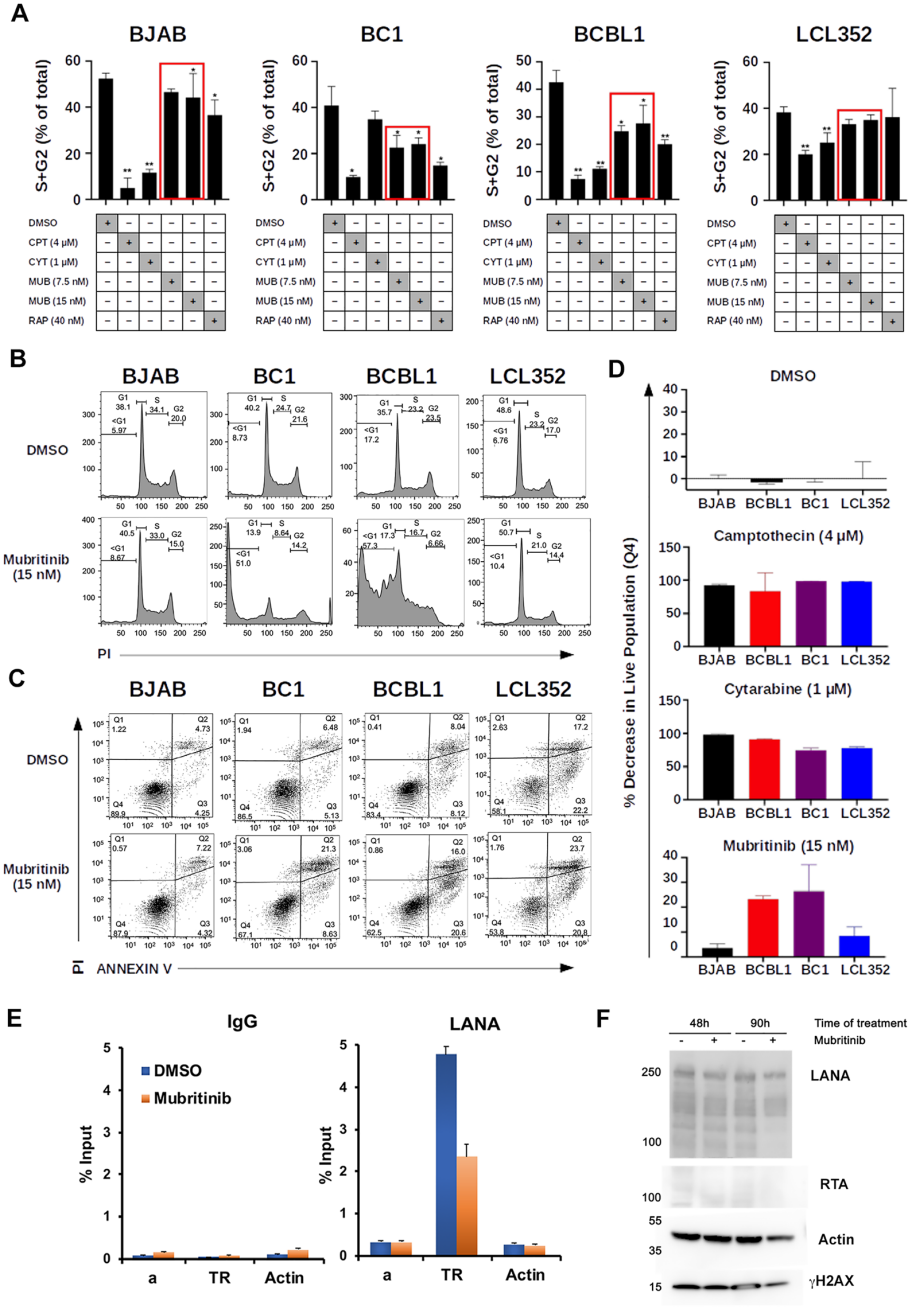
Mubritinib inhibits PEL cell cycle progression and LANA DNA binding. (**A**) Graph of cell cycle kinetics data comparing Mubritinib (MUB) to cytarabine (CYT) and rapamycin (RAP). DMSO and camptothecin (CPT) were used as controls. PEL cells (BC1 and BCBL1) treated with 7.5 nM Mubritinib show a significant decrease in the total population of S and G2 cells that is not observed for BJAB or LCL352 cells. Neither cytarabine nor rapamycin showed similar selectivity for inhibition of PEL cell growth (S, G2) (^**^ *p* < 0.001, ^*^ *p* < 0.05; Student’s *T* Test). (**B**) Cell cycle profiles comparing cells (BJAB, BC1, BCBL1, and LCL352) treated with DMSO and 15 nM Mubritinib measured by FACS flow cytometry analysis of propidium iodide staining. (**C**) Flow cytometry analysis of Annexin V/PI staining comparing cells (BJAB, BC1, BCBL1, LCL352) treated with DMSO and 15 nM Mubritinib. (**D**) Graphs summarizing the decrease in live cell populations observed from the Annexin V/PI experiment. Camptothecin (4 mM) and Cytarabin (1 mM) are shown for comparison. (^**^ *p* < 0.001; Mann–Whitney). (**E**) ChIP-qPCR assay for LANA or IgG control in BCBL1 cells treated with Mubritinib (15 nM) for 72 hrs with primers for KSHV TR, control region a, or cellular Actin. (**F**) Western blot control of ChIP assays showing LANA, RTA, Actin, or gH2AX in BCBL1 cells at 48 h after addition of DMSO (–) or Mubritinib (15 nM) (+).

After identifying Mubritinib as a hit, we sought to compare the selectivity that we observed with this drug to other published drugs that have been tested against PEL cell lines. In particular, we chose to compare Mubritinib to cytarabine (CYT) [35] and rapamycin (RAP) [36]. We began by testing the effects of these drugs on the cell cycle using propidium iodide staining and flow cytometry (Figure 4A and 4B, Supplementary Figure 1). For these experiments, we chose to test each drug at concentrations similar to those that have been previously published [35, 36]. DMSO and camptothecin (CPT) were used as negative and positive controls, respectively. When BJAB and LCL352 cells were treated with 7.5 nM Mubritinib, the total population of S and G2 cells was similar to that observed when these cells were treated with DMSO (Figure 4D). However, when PEL cells (BC1 and BCBL1) were treated with the same concentration of Mubritinib, a significant decrease in the total population of S and G2 cells (Figure 4A), and corresponding increase in G1+subG1 (Supplementary Figure 1) was observed. In contrast, neither cytarabine nor rapamycin showed selective inhibition of KSHV^+^ PEL cell growth as did Mubritinib (Figure 4A and Supplementary Figure 1).

### Mechanism of action of Mubritinib in PEL cells

To begin to understand the mechanism of action of Mubritinib, we first analyzed the cell cycle profiles for two KSHV^+^ PELs (BC1 and BCBL1) and two KSHV^-^ lymphomas (BJAB and LCL352) after treatment with 15 nM Mubritinib (Figure 4B). Mubritinib treatment altered the cell cycle profiles of BC1 and BCBL1 cells, while having no detectable effects on the cell cycle profiles of BJAB or LCL352 cells. For the two PEL cell lines (BC1 and BCBL1), Mubritinib reduced S and G2 populations and increased the sub-G_1_ population (Figure 4B). To further investigate these effects on cell viability markers, we next performed flow cytometry with Annexin V/PI binding analysis (Figure 4C and 4D). Treatment with 15 nM Mubritinib caused higher decreases in live and increases in apoptotic cell populations for the PEL cell lines (BC1 and BCBL1) compared to BJAB and LCL352 cells (Figure 4D). We also compared the effects of Mubritinib with cytarabine and camptothecin (Figure 4D). Only Mubritinib, and not cytarabine or camptothecin, showed strong selectivity for PEL cells when scored for percentage of live cells.

To assess whether Mubritinib can inhibit the binding of LANA to KSHV genomes, we performed chromatin-immunoprecipitation (ChIP) assays in BCBL1 cells (Figure 4E). We found that LANA binding to the TR was reduced ~50% in BCBL1 cells treated with 15 nM Mubritinib for 72 hrs. LANA did not bind to negative control regions of the viral or cellular genome, indicating specificity in the LANA ChIP assay. Furthermore, Mubritinib did not induce lytic activator RTA at either 48 or 90 hrs of treatment, as measured by Western blot analysis (Figure 4F) or RT-qPCR of KSHV lytic cycle genes (Supplementary Figure 2). Interestingly there was a modest down regulation of LANA by Western blot (Figure 4F) and ORF73 mRNA expression as measured by RT-PCR (Supplementary Figure 2) after 48 hours treatment of BCBL1 cells. Taken together, these findings indicate that nanomolar concentrations of Mubritinib cause cell cycle arrest with accumulation of sub-G_1_ cell populations selective for KSHV^+^ PEL cells, and partially disrupt LANA binding to TR.

### Mubritinib also exhibits selectivity *in vivo*

**Figure 5:**
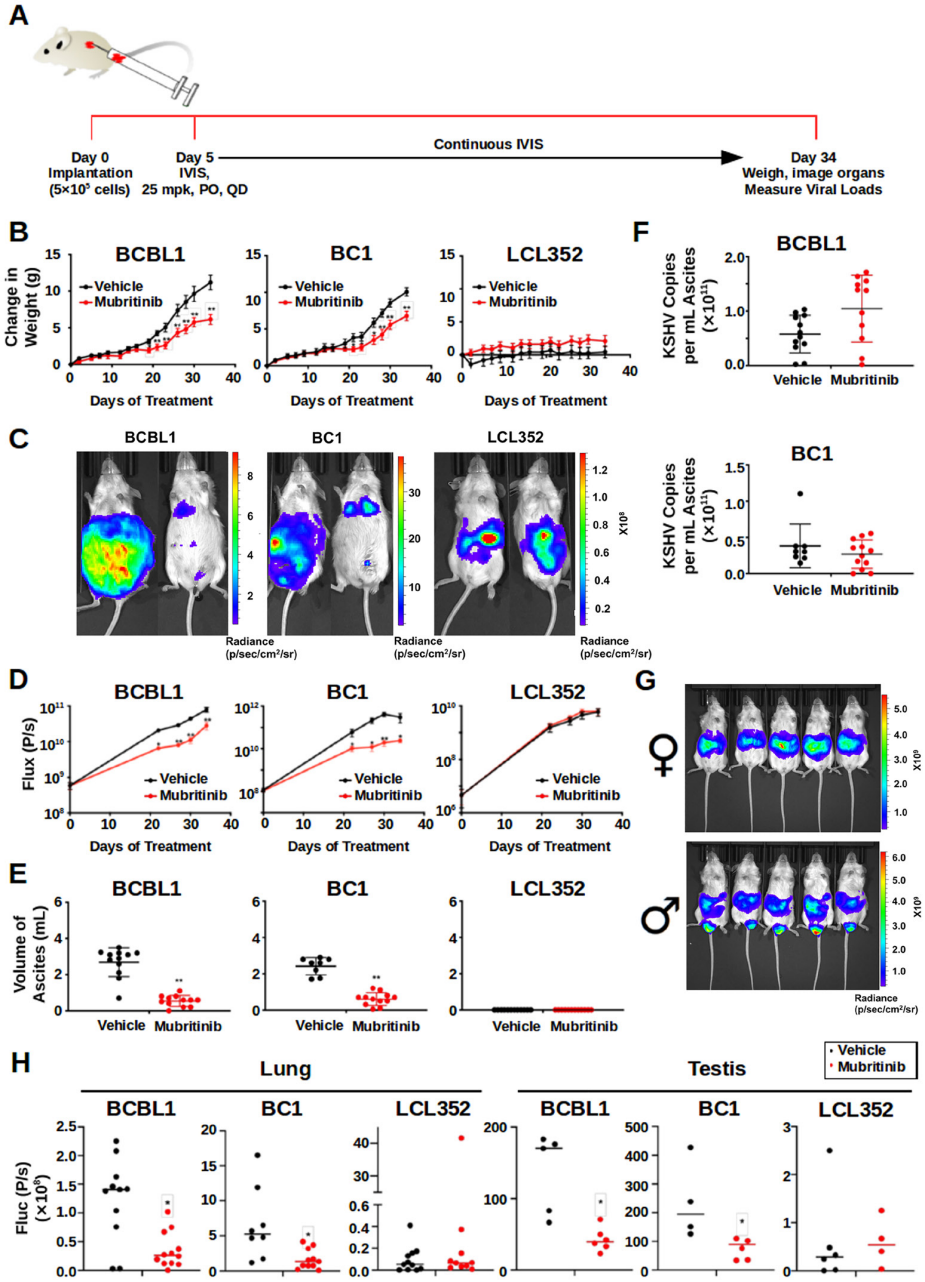
Mubritinib Inhibits PEL tumor growth in mouse xenografts. (**A**) Cartoon illustrating key features of the *in vivo* experiments that were performed with Mubritinib. NSG mice were implanted with 5 × 10^5^ BCBL1, BC1, or LCL352 cells intraperitoneally on Day 0. Beginning on Day 5, the mice were treated daily with 0 (vehicle) or 25 mg/kg (mpk) Mubritinib orally. During treatment, the mice were continuously imaged using a Spectrum IVIS CT Bioluminescent Imaging System. At Day 34, the mice were sacrificed, weighed, and selected organs were collected for further analyses. (**B**) Graphs comparing the changes in weight that were observed in mice implanted with PEL cell lines (BCBL1 and BC1) versus mice implanted with the EBV^+^ LCL352 B-cell line (^*^ *p* < 0.05, ^**^ *P* < 0.01; *T* test). (**C**) Images comparing the bioluminescence signals that were observed in mice implanted with PEL cell lines (BCBL1 and BC1) versus mice implanted with LCL352 cells. For each pair, the left mouse was treated with vehicle and the right mouse was treated with Mubritinib. (**D**) Graphs showing the changes in bioluminescence flux (photons/s) over time. These results are in agreement with those observed in (C) (^**^ *P* < .01; *T* test). (**E**) Graphs showing the volumes of ascites that were collected at the end of the *in vivo* experiments. (^**^ *p* < 0.0001; Mann–Whitney). Notably, animals engrafted with LCL352 cells did not develop ascites. (**F**) Graphs showing the number of KSHV copies per mL of ascites. (**G**) Images comparing the bioluminescence signals from female (top) and male (bottom) mice. The male mice developed a strong bioluminescence signal in their testis. (**H**) Graphs showing the bioluminescence flux (photons/s) in the lungs and testis in vehicle (black) vs Mubrtinib (red) (^*^ *p* < 0.01; Mann–Whitney).

Given the potency and selectivity that Mubritinib displayed for inhibition of PEL cell growth in culture, we decided to examine the effects of this drug in two PEL mouse models (Figure 5A). For comparison, we chose to implant mice with EBV^+^ LCL352 B-cells. All of the xenografts were transduced with mCherry/eLuciferase for *in vivo* imaging. BCBL1 and BC1 mice treated with vehicle rapidly gained weight due to xenograft growth (Figure 5B). However, this increase was significantly slowed in mice treated once daily with 25 mg/kg Mubritinib. By comparison, LCL352 mice treated with Mubritinib exhibited weights similar to those treated with vehicle. All of the mice that were treated with vehicle developed strong bioluminescence signals (Figure 5C and 5D). However, only the BCBL1 and BC1 mice treated with Mubritinib demonstrated a reduction in bioluminescence signals when compared to their vehicle controls.

This inhibition of PEL cell growth *in vivo* was further validated by measuring ascites volumes (Figure 5E). Mubritinib treatment caused a significant reduction in the volumes of ascites for the BCBL1 and BC1 xenograft mice. LCL352 mice did not develop large volumes of ascites when they were treated with either vehicle or Mubritinib. Interestingly, although the total volume of ascites was reduced for the BCBL1 and BC1 mice upon treatment with Mubritinib (Figure 5E), the KSHV copies per mL of ascites for these mice remained similar to the corresponding vehicle treated controls (Figure 5F). This suggests that, at least in these *in vivo* experiments, Mubritinib had little effect on KSHV episome copy number. This is consistent with our previous observation that Mubritinib inhibits PEL cell growth at concentrations far lower than those that were necessary to disrupt LANA DNA binding (Figures 2 and 3).

For our *in vivo* experiments, we used both female and male mice (Figure 5G). And, in general, the localization of the bioluminescence signals that we observed were similar for mice of both genders. However, the male mice also developed strong signals in their testes. Interestingly, the intensities of these signals were far greater in the BCBL1 and BC1 mice than the LCL352 mice (Figure 5H). Furthermore, the bioluminescence signals in the testes of the BCBL1 and BC1 mice were significantly reduced when the mice were treated with Mubritinib. Similarly, the bioluminescence signals in the lungs of the BCBL1 and BC1 mice were significantly reduced when they were treated with Mubritinib (Figure 5H). Collectively, these data confirm that Mubritinib is a potent inhibitor of PEL cell growth *in vivo* and that its effects are selective when compared to EBV^+^ LCL352 B-cells.

### Mubritinib does not act as a HER2/ErbB2 inhibitor in PEL cells

Mubritinib (also known as TAK 165) was originally identified as a potent and selective inhibitor of receptor tyrosine kinase HER2/ErbB2 [37]. Subsequently, Grygielewicz et al. later demonstrated that SNU-16 gastric cancer cells that had become resistant to fibroblast growth factor receptor (FGFR) inhibitors became sensitive to treatment with Mubritinib [38]. Interestingly, the development of resistance to FGFR inhibitors in these cells was accompanied by a dramatic reduction in expression of various tyrosine kinase receptors, including HER2/ErbB2. Therefore, it was proposed that Mubritinib acted by a mechanism that does not involve inhibition of HER2/ErbB2. More recently, Baccelli et al. provided compelling evidence that Mubritinib selectively inhibits growth of a subset of acute myeloid leukemia (AML) cells that rely on mitochondrial oxidative phosphorylation (OXPHOS) and anaerobic glycolysis [39]. They determined that the molecular target for Mubritinib in these cells was the electron transport chain (ETC) complex I.

**Figure 6:**
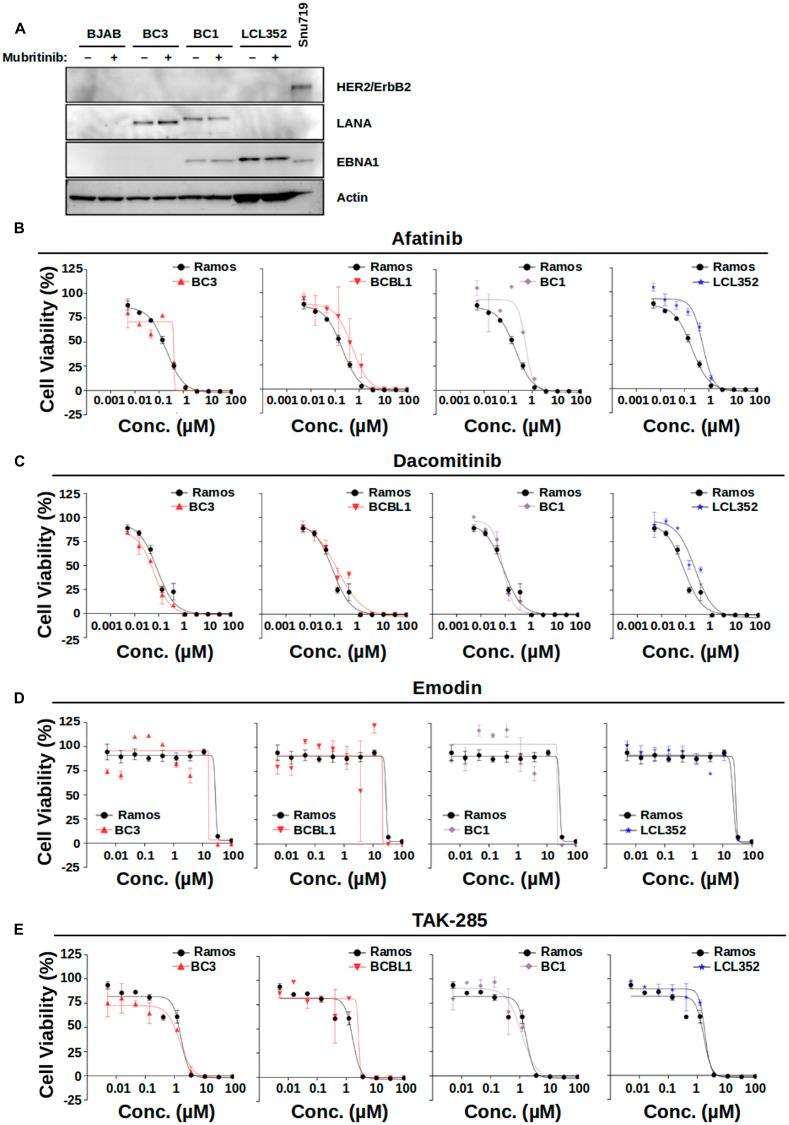
HER2 inhibitors are not selective for PEL cell growth inhibition. (**A**) Western blot demonstrating the level of HER2/ErbB2 expression in various cell lines. SNU-719 gastric cancer cells were used as a positive control. Expression of LANA was used to confirm infection with KSHV. Expression of EBNA1 was used to confirm infection with EBV. Actin was used as a loading control. (**B**–**E**) Titration of published HER2/ErbB2 inhibitors (B) Afitinib, (C) Dacomitinib, (D) Emodin, and (E) TAK-286 against various B-cell lines. Ramos (KSHV^-^, EBV^-^) data are shown for reference in each graph. None of these HER2/ErbB2 inhibitors display the same selectivity for PEL cell growth inhibition as Mubritinib shown in Figure 3E.

To clarify the mechanism of action for Mubritinib in PEL cells, we began by examining the level of HER2/ErbB2 expression in these cells (Figure 6A). As a positive control, we also examined SNU-719 gastric cancer cells. However, we were not able to observe HER2/ErbB2 protein in PEL cells by Western blot. Similarly, we did not detect HER2/ErbB2 in lysates from KSHV^-^ BJAB cells or EBV^+^ LCL352 cells. These results suggest that inhibition of PEL cell growth by Mubritinib may not involve inhibition of HER2/ErbB2. To further investigate this, we analyzed a series of published HER2/ErbB2 inhibitors, namely Afatinib, Dacomitinib, Emodin, and TAK-285 (Figure 6B–6E, Supplementary Table 2). However, none of these inhibitors displayed the same selectivity as Mubritinib (Figures 2E and 3G). These findings suggest that Mubritinib does not act as a HER2/ErbB2 inhibitor in PEL cells.

### ETC complex I inhibitors exhibit selectivities similar to Mubritinib for inhibition of PEL cell growth

**Figure 7:**
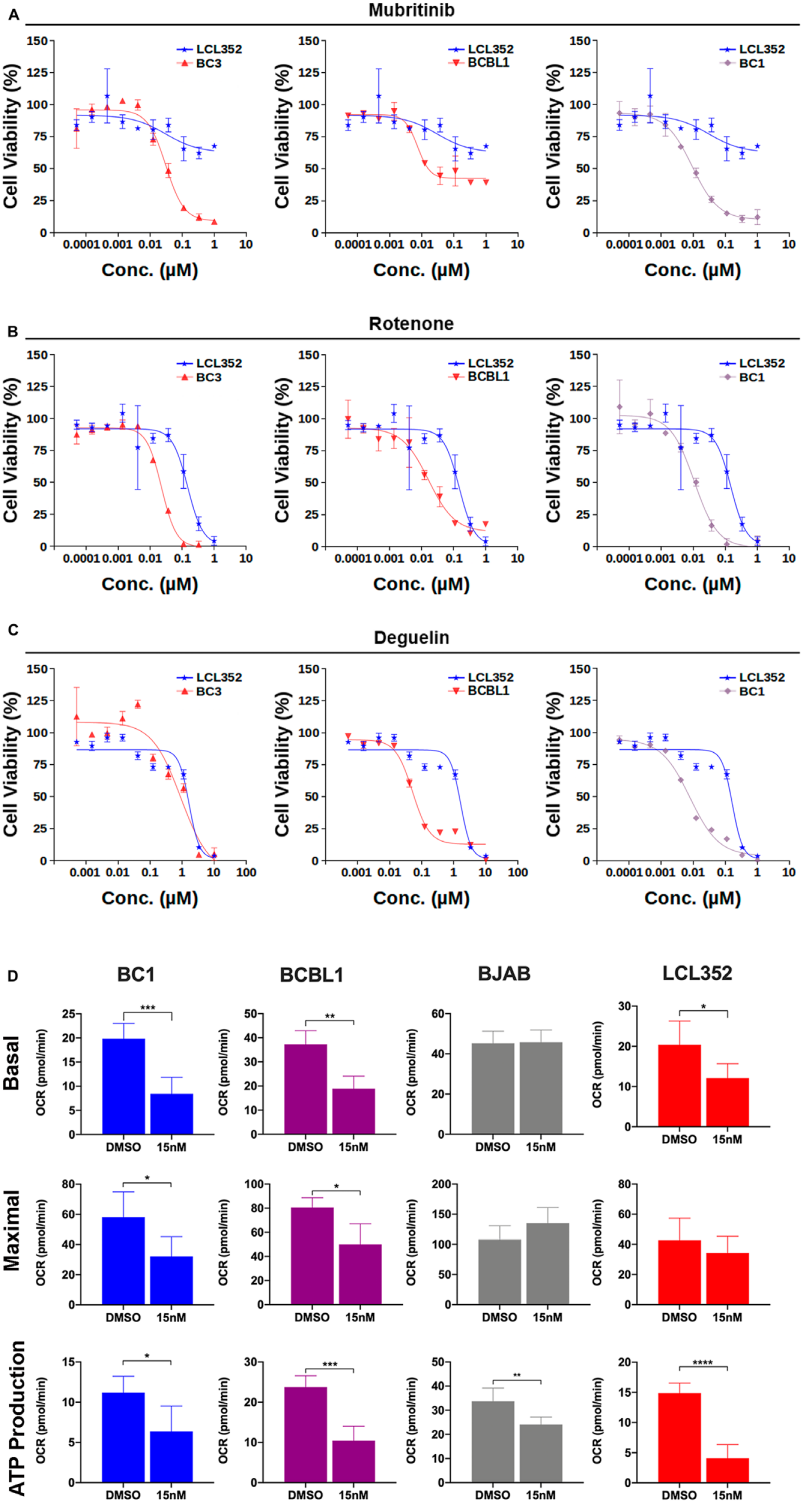
Electron transport chain (ETC) complex I inhibitors exhibit selectivities similar to Mubritinib for inhibition of PEL cell growth. (**A**) Titration of Mubritinib against various B-cell lines. EBV^+^ (LCL352) data (blue) are shown for reference in each graph. KSHV^+^ PEL cell lines (BC3, BCBL1, and BC1) respond more strongly to Mubritinib at nanomolar concentrations. (**B**) Titration of Rotenone, an ETC complex I inhibitor, against the same B-cell lines shown in (A). (**C**) Titration of Deguelin, an ETC complex I inhibitor, against the same B-cell lines shown in (A). (**D**) Effects of Mubritinib on basal and maximal oxygen consumption rates (OCR) as well as ATP production for each cell line. (^***^ *p* < 0.0001, ^**^ *p* < 0.001, ^*^ *p* > 0.05; *T*-test).

Given recent literature that suggests that Mubritinib can inhibit the electron transport chain (ETC) complex I [39], we next investigated whether published ETC complex I inhibitors exhibit similar selectivity for growth inhibition of PEL cells (Figure 7A–7C, Supplementary Table 2). Rotenone, like Mubritinib, displayed clear selectivity for inhibition of PEL cell growth at nanomolar concentrations (Figure 7B). Deguelin also exhibited partial selectivity for BCBL1 and BC1 PEL cell lines (Figure 7C). These results suggest that PEL cells are selectively sensitive to drugs targeting the ETC. More detailed analysis of the impact of basal and maximal oxygen consumption rates (OCR) and basal ATP production was measured by Seahorse analysis (Figure 7D and Supplementary Figure 3). This revealed that Mubritinib inhibits ATP production in all cells tested, but that maximal and basal OCR levels were more selectively inhibited in KSHV+ PEL cells relative to LCLs and gammaherpesvirus negative BJABs. The maximal respiratory rate is determined by several factors, including the functional capacity of the electron transport chain. Therefore, these results suggest that disruption of maximal OCR by Mubritinib is specific to KSHV+ cells and that this disruption of OXPHOS prevents the electron transport chain in KSHV+ cells from operating at maximum capacity.

### Mubritinib selectively alters key metabolite levels in PEL cells

**Figure 8:**
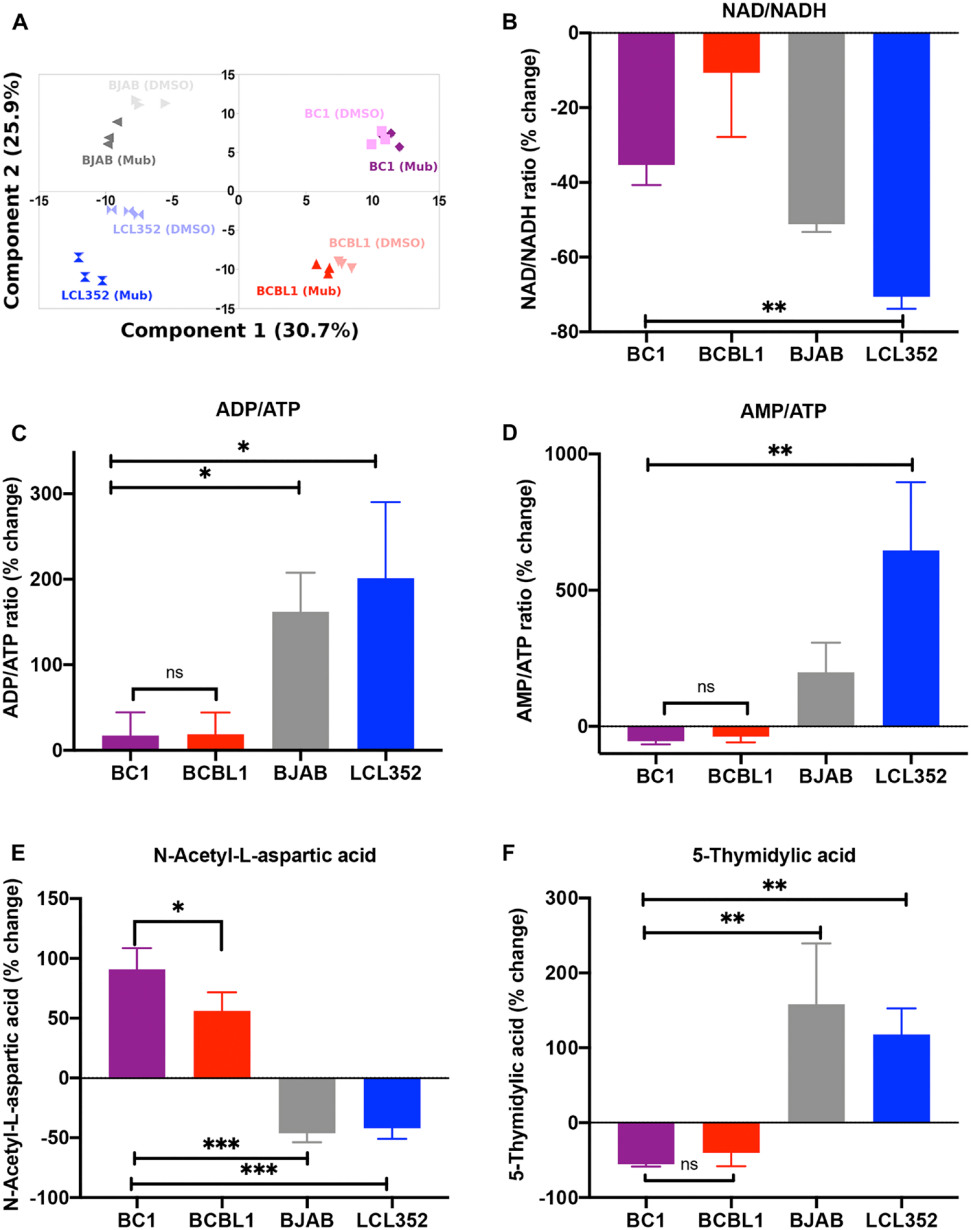
Mubritinib selectively alters key metabolite levels and impairs the electron transport chain in PEL cells. (**A**) Principle component analysis (PCA) for the metabolomics data. These results confirm that sample replicates cluster together based on cell line (BJAB, LCL352, BC1, BCBL1) and treatment condition (DMSO, Mubritinib). (**B**) Percent change in NAD/NADH ratios. (**C**) Percent change in AMP/ATP ratio observed upon treatment with Mubritinib (relative to DMSO treatment) for each cell line. (**D**) Percent change in ADP/ATP ratios. (**E**) Percent change in N-acetyl-L-apartic acid levels (relative levels determined by normalized peak area). (**F**) Percent changes in 5-thymidylic acid levels (relative levels determined by normalized peak area). (^***^ *p* < 0.001, ^**^ *p* < 0.01, ^*^ *p* > 0.05; One-Way-Anova, post-hoc analysis by Tukey test).

In AML cells, inhibition of ETC complex I by Mubritinib was accompanied by specific changes in the levels of various metabolites [39]. To assess the effect of Mubritinib on metabolites in PEL cells, we performed a global metabolomic analysis by mass spectrometry for a panel of B-cells, including KSHV^+^ BC1 and BCBL1, and KSHV^-^ BJAB and LCL352 (Figure 8A). As with AML cells [46], we observed that Mubritinib reduced the NAD/NADH ratio in KSHV^+^ PEL cells (Figure 8B). However, we also saw a reduction in the NAD/NADH ratio for a KSHV^-^/EBV^-^ cell line (BJAB) and a KSHV^-^/EBV^+^ cell line (LCL352). Therefore, we concluded that this reduction may not explain selectivity that Mubritinib exhibits for PEL cell growth inhibition. Conversely, Mubritinib treatment produced distinctly unique responses in ADP/ATP (Figure 8C) and AMP/ATP (Figure 8D) ratios in PEL cells. Moreover, N-acetyl-L-aspartic acid (Figure 8E) and 5-thymidylic acid (Figure 8F) levels were also uniquely altered upon treatment with Mubritinib. Interestingly, although Baccelli et al. observed that Mubritinib treatment led to a decrease in aspartate levels in AML cells, we observed an increase in N-acetyl-L-aspartic acid in PEL cells. In contrast, we observed a decrease in N-acetyl-L-aspartic acid for the two KSHV^-^ cell lines (BJAB and LCL352). These findings suggest that Mubritinib selectively alters metabolic pathways and the ETC in ways that contribute to the selective inhibition of KSHV positive PEL cell growth.

## DISCUSSION

KSHV associated cancers remain an unmet medical need due to their high rates of resistance to existing chemotherapies [21]. PEL patients typically have less than 1 yr survival time even with aggressive chemotherapy [40]. Many other drugs and natural products have been reported to inhibit PEL cell growth and tumorigenesis [35, 41–55]. However, even though KSHV is known to drive this cancer, at present there are no inhibitors that selectively and effectively target KSHV gene products.

In this work, we focused on identification of known drugs that could be readily repurposed for the treatment of PEL. We screened a small collection of known drugs from SelleckChem and identified Mubritinib as an interesting candidate based on its ability to selectively inhibit LANA DNA binding and selectively inhibit KSHV^+^ PEL cell growth *in vitro* and *in vivo*. Remarkably, cells infected with EBV, a γ-herpesvirus that is closely related to KSHV, responded similar to other KSHV^-^ cells when treated with Mubritinib.

Our findings indicate that Mubritinib can inhibit LANA DNA binding in a cell-based high-throughput assay (Figure 2) and in ChIP assays in PEL cells (Figure 4). Previous studies demonstrated that prolonged knockdown of the KSHV protein LANA in a PEL cell line led to the loss of KSHV episomes [56]. Similarly, CRISPR deletion of LANA led to a loss of KSHV genomes from PEL cells [33]. In these genetic disruptions, LANA depletion did not lead to cell cycle arrest or loss of viability. Although complete loss of KSHV genomes may not have been achieved, these studies raise the concern that loss of LANA and KSHV genomes may not be sufficient to block PEL tumorigenesis. Thus, it is likely that Mubritinib inhibition of PEL cell growth is due, at least in part, to activities other than LANA inhibition.

While Mubritinib was originally identified as a HER2/ErbB2 inhibitor [37], our findings suggest that its selectivity for PEL growth inhibition is dependent on its additional activity in blocking electron transport and mitochondrial metabolism [39]. Metabolic processes were recently determined to be among the top hits in a CRISPR screen for essential pathways in PEL cells [57]. In endothelial cells, KSHV is known to induce the Warburg effect, a cancer-associated metabolic shift to aerobic glycolysis [58, 59]. Yet, suppression of glycolysis is observed during infection of mesenchymal stem cells [60]. KSHV can induce nuclear factor erythroid2-related factor 2 (NRF2) [61], a regulator of cellular resistance to oxidants, and reactive oxygen species (ROS) during endothelial cell infection [62]. Furthermore, inhibitors of ROS were shown to block mouse models of KS [62]. mTOR inhibitors induce mitochondrial apoptosis in PEL and KS, and the mechanism may be related to disruption of oxidative phosphorylation, similar to Mubritinib and Rotenone [63, 64]. We found that Mubritinib selectively inhibited the maximal OCR in KSHV^+^ PEL relative to other B-cell lines (Figure 7D), as well as the ADP/ATP (Figure 8C) and ATP/AMP (Figure 8B) ratios. We also found Mubritinib selectively altered N-acetyl-aspartic acid (Figure 8E) and 5-thymidylic acid (Figure 8F) abundance in KSHV^+^ PEL cells. These findings are consistent with Mubritinib inhibition of the ETC as a mechanism of selective inhibit PEL cell growth.

In conclusion, we provide evidence that Mubritinib can inhibit LANA DNA binding and PEL cell growth. The selective inhibition of PEL cells correlated with inhibition of mitochondrial OXPHOS function. The mechanism of inhibition of LANA DNA binding by Mubritinib, as well as other RTK inhibitors, is not yet know. It is possible that dual activities of Mubritinib may enhance its selective inhibition of KSHV+ PEL cells. In this work, we focused on the mechanism of inhibition of PEL cell growth and found a vulnerability in PEL cell mitochondrial OXPHOS function. These findings may inform development of more selective and efficacious treatments for PEL and other KSHV-associated cancers. Preclinical studies for Mubritinib in a murine model of bladder cancer were performed at doses of 10 mg/kg and 20 mg/kg administered BID (twice daily) [37]. Therefore, our study showing an effect of Mubritinib at a similar dose (25 mg/kg), administered only once daily suggests that we are well within a dose range that is consistent with studies used for other cancer indications. Moreover, the paucity of specific treatments available for PEL underscores the potential importance of our *in vivo* findings.

## MATERIALS AND METHODS

### Chemicals

The SelleckChem library from the Molecular Screening Facility at the Wistar Institute was used for initial screens. However, once hits were identified, additional material was purchased for further testing. Mubritinib (S2216), Carmofur (S1289), Erlotinib HCl (S1023), NSC-207895 (S2678), OSI-420 (S2205), Emodin (S2295), TAK-285 (S2784), and Deguelin (S8132) were purchased from SelleckChem. Linifanib (M15676), Regorafenib (R16040), and Afatinib (G-7208) were purchased from AChemBlock. Adrucil (228440010) and Cytarabine (449561000) were purchased from Acros Organics. Lenalidomide (6305/100) and Dopamine HCl (3548/50) were purchased from Tocris Bioscience. Dacomitinib (PZ0330) and Rotenone (R8875) were purchased from Sigma. Rapamycin (J62473) was purchased from Alfa Aesar.

### Antibodies

The following antibodies were used for Western blot analysis: rabbit β-tubulin (Cell Signaling Technology, 2146S), goat anti-rabbit IgG (H+L)-HRP Conjugate (Bio-Rad, 170-6515), ANTI-FLAG M2-Peroxidase (Sigma Aldrich, A8592), Apoptosis Western Blot Cocktail (abcam, ab136812), rat anti-LANA (Advanced Biotechnologies Inc., 13210), mouse anti-EBNA1 (Biorad), rabbit anti-HER2 (D8F12) (Cell Signaling), mouse monoclonal anti-actin-HRP (Sigma).

### Plasmids

The *Gaussia* luciferase reporter plasmid was generated by removing the firefly luciferase sequence from the pGL4.31 plasmid using FseI and HindIII-HF enzymes (New England Biolabs) and replacing it with the *Gaussia* luciferase sequence. Then, two complementary oligonucleotides containing an AflII restriction site were inserted upstream of the MLP promoter using EcoRI-HF and NheI enzymes (New England Biolabs). Finally, complementary oligonucleotides containing the three LANA DNA binding sites (LBS2-LBS1-LBS3) were inserted upstream of the MLP promoter using AflII and NheI enzymes (New England Biolabs). The RMCE-HILO donor plasmid was generated by digesting the pEM791 plasmid [65] with AgeI-HF and BsrGI-HF enzymes (New England Biolabs) and inserting two PCR amplified sequences containing the FLAG tag fused to the VP16 activation domain and the LANA DNA-binding domain by Gibson Assembly (New England Biolabs).

### Cell lines

The RMCE-HILO HEK293T acceptor cells were obtained from Eugene V. Makeyev (Nanyang Technological University, Singapore) and maintained in Dulbecco’s modified Eagle’s medium (Gibco BRL) with 10% FetalPlex Serum Complex (Gemini Bio) and penicillin and streptomycin (50 U/ml). Ramos cells (uninfected Burkitt Lymphoma from ATCC CRL-1596) and BJAB (uninfected B cell lymphoma) cells were obtained from ATCC and maintained in RPMI medium (Gibco BRL) containing 10% heat-inactivated fetal bovine serum and penicillin and streptomycin (50 U/ml). KSHV^+^ single positive PEL cells (BCBL1 and BC3) and double positive KSHV and EBV infected PEL cells (BC1) were provided by Yan Yuan (University of Pennsylvania) and maintained in RPMI medium (Gibco BRL) containing 10% heat-inactivated fetal bovine serum and penicillin and streptomycin (50 U/ml).

### Gaussia luciferase screens

On the first day, 4–5 × 10^6^ HEK293T acceptor cells were seeded in a 10-cm plate. The following day, the media was replaced with antibiotic-free media and the cells were transfected with 3 μg reporter plasmid using Lipofectamine 2000 (Invitrogen) at a ratio of 1:3 (plasmid: transfection reagent). Approximately 24 hours after transfection, the cells were trypsinized, counted, and diluted to 150,000 cells/mL using Dulbecco’s modified Eagle’s medium (Gibco BRL) with 2.5% FetalPlex Serum Complex (Gemini Bio) and penicillin and streptomycin (50 U/ml). These diluted cells were separated into two aliquots (–dox and +dox) and doxycycline was added to one of the aliquots to a final concentration of 0.125 μg/mL. Then, 40 μL of cells were seeded into each well of a 384-well plate. Control wells were treated with DMSO at a final concentration of 0.125%. The remaining wells were treated with drugs from the SelleckChem library (dissolved in DMSO) at a final concentration of 12.5 μM. Approximately 24 hours after addition of the drugs, the *Gaussia* luciferase signal for each well was measured (BioLux Gaussia Luciferase Assay Kit, New England Biolabs) using an Envision Plate Reader. Then, the cells were incubated with resazurin (Sigma) at a final concentration of 50 μM for ~4 hours and the cell viability was measured using an Envision Plate Reader. The % luciferase signal for each drug was calculated relative to the –dox value (0%) and the +dox value (100%). The % cell viability for each drug was calculated relative to complete inhibition of cell viability (0%) and treatment with DMSO (100%).

### B-cell viability screens

BC3 and Ramos cells were counted and diluted to 100,000 cells/mL and 150,000 cells/mL, respectively, with RPMI medium (Gibco BRL) containing 2.5% heat-inactivated fetal bovine serum and penicillin and streptomycin. Each cell line was separated into two aliquots (–puro and +puro) and puromycin (Sigma-Aldrich) was added to one of the aliquots. Then, 50 μL of cells were seeded into each well of a 384-well plate. Control wells were treated with DMSO at a final concentration of 0.1%. The remaining wells were treated with drugs from the SelleckChem library (dissolved in DMSO) at a final concentration of 10 μM. Approximately 72 hours after addition of the drugs, the cells were incubated with resazurin (Sigma) at a final concentration of 50 μM for ~4 hours and the cell viability was measured using an Envision Plate Reader. The % cell viability for each drug was calculated relative to the +puro value (0%) and treatment with DMSO (100%).

### B-cell hit titrations

Cells were diluted with RPMI containing 2.5% heat-inactivated fetal bovine serum and penicillin and streptomycin as follows: Ramos (120,000 cells/mL), BC3 (160,000 cells/mL), BCBL1 (80,000 cell/mL), BJAB (12,000 cells/mL), BC1 (100,000 cells/mL), LCL352 (40,000 cells/mL). Then, cells were seeded at a final volume of 50 μL into a 384-well plate and treated with drugs dissolved in DMSO. The final concentration in the well was 0.1%. Wells treated with DMSO or puromycin (+puro) were used as controls. Approximately 72 hours after addition of the drugs, the cells were incubated with resazurin (Sigma) at a final concentration of 50 μM for ~4 hours and the cell viability was measured using an Envision Plate Reader. The % cell viability for each drug was calculated relative to the +puro value (0%) and treatment with DMSO (100%).

### Analysis of cell cycle kinetics

BC1 (KSHV^+^, EBV^+^), BCBL1 (KSHV^+^, EBV^-^), and LCL352 (KSHV^-^, EBV^+^) cells were seeded in 6-well plates and exposed to camptothecin (4 μM), rapamycin (40 nM), cytarabine (1 μM), or Mubritinib (15 nM and 7.5 nM) in biological triplicates per each condition. After 72 h, cells were permeabilized with cold, 70% ethanol and resuspended in PBS containing PI (10 mg/mL) and RNAse A solution (100 μg/mL). Flow cytometry was performed on a BD-LSR II (BD Biosciences; Bedford, MA, USA) and data were analyzed using FloJo software (Ashland, OR, USA).

### Annexin V/PI binding assay

An AnnexinV-FITC apoptosis detection kit (Abcam; Cambridge, UK) was used to confirm the effect of Mubritinib and other compounds of interest on cell viability. BC1 (KSHV^+^, EBV^+^), BCBL1 (KSHV^+^, EBV^-^), and LCL352 (KSHV^-^, EBV^+^) cells were seeded in 6-well plates and exposed to camptothecin (4 μM), rapamycin (40 nM), vargatef (40 nM), AST-1306 (10 nM), cytarabine (1 μM), or Mubritinib (15 nM and 7.5 nM) in biological triplicates per each condition. After 72 h, the percentages of live and apoptotic cells were analyzed after double staining the cells with FITC conjugated Annexin V and propidium iodide (PI). Flow cytometry was performed on a BD-LSR II (BD Biosciences; Bedford, MA, USA) and data were analyzed using FloJo software (Ashland, OR, USA).

### Ethics statement

All animal experiments were conducted under the Wistar Institute’s approved Institutional Animal Care and Use Committee Protocol #201158 in accordance with the Committee for the Purpose of Control and Supervision of Experiments on Animals guidelines for animal experimentation. All mice in this study were managed in accordance with the NIH Office of Laboratory Animal Welfare: “PHS Policy on the Humane Care and Use of Research Animals”; the recommendations of the American Association for Accreditation of Laboratory Animal Care (AAALAC).

### Mice

NSG mice (NOD. Cg-*Prkdc^scid^ Il2rg^tm1Wjl^*/SzJ) were bred in-house at The Wistar Institute under protocol #112092. Mice were enrolled at 6–8 weeks of age and housed in micro-isolator cages in a designated, specific pathogen-free facility where they were fed sterile food and water *ad libitum*. Mice were euthanized via CO_2_ administration according to AALAC euthanasia guidelines.

### Tumor implantation, grouping, and equalization

Mice (six males and six females per treatment group) were engrafted with a cell suspension (>98% viability) of 5 × 10^5^ BC1-mCherry/eLUC (KSHV+, EBV^+^), 5 × 10^5^ BCBL1-mcherry/eLUC (KSHV^+^, EB V^-^), or 5 × 10^5^ LCL352- mCherry/eLUC (KSHV^-^, EBV^+^) cells resuspended in 1× PBS, pH 7.4 and maintained on ice. Animals were weighed three-times per week and monitored daily. The Spectrum IVIS CT Bioluminescent Imaging System (Perkin-Elmer; Waltham, MA) was used to randomize mice into groups of 12 per treatment (six males and six females) on day five so ensure the average Flux (Photons/sec) was equivalent across groups and to monitor cell growth and metastasis throughout the study. For imaging studies, mice were injected with D-luciferin (Gold Biotechnology), i.p. at a dose of 7.5 mg/kg in a dose volume of 10 ml/kg body weight 15 minutes prior to imaging; this was the optimal interval between luciferin injection and bioluminescent imaging as determined by an initial kinetic curve for these cell lines in mice. Mice were anesthetized using isoflurane prior to imaging. Total body Flux (Photons/Sec) was quantitated throughout the study using the IVIS imaging software. Flux measurements for the liver, spleen, kidneys, heart, lungs, and testis were obtained postmortem at the terminal timepoint on day 34.

### Mubritinib formulation and treatment schedule

Mubritinib was weighed and vehicle was added immediately prior to dose. The vehicle was 10% DMSO, 2.5% Tween^®^ 80 + 97.5% of 0.5% Carboxy Methyl Cellulose (CMC). The vehicle control comprised formulation reagents without compound. Mubritinib was administered q. d., p. o. at 25 mg/kg in a dose volume of 10 ml/kg body weight.

### Quantitative PCR for ascites viral load

Viral loads were quantified with a standard curve using quantitative PCR. Standards were prepared using a full-length KSHV genome bacterial artificial chromosome, BAC16 (1). Using a determined DNA concentration and size of the KSHV genome (138 Kbp), we calculated BAC16 copy number per microliter. We then prepared a series of six 10-fold dilution standards of known concentration and copy number from the isolated BAC16 DNA stock. Total DNA was extracted from ascites fluid using the DNeasy Blood and Tissue kit (Qiagen; Germantown, MD, USA). All DNA samples were plated in triplicate on a 384 well plate. Each well contained 5 μl of sample and 10 μl of master mix. The master mix in each well included 7.5 μl of SYBR green, 0.15 μl of each 10 μmol primer for ORF50 (Sequences: 5′-CCCGCCCAGAAACCAGTAG-3′ and 5′-TGCGGAGTAAGGTTGACTTTTTAA-3′), and 2.2 μl of ddH20. Samples were amplified using Applied Biosystems 7900HT Fast Real Time PCR machine. Each cycle consisted of four stages: 1) 50°C for 2 minutes; 2) 95°C for 10 minutes; 3) 95°C for 15 seconds followed by 60°C for 1 minute; 4) 95°C for 15 seconds followed by 60°C for 1 minutes. Ct values were plotted against the logarithm of copy number for BAC16 standards to generate a regression line (R^2^ > 0.99). The regression equation was used to determine copy numbers for each sample.

### Quantitative PCR for KSHV mRNA expression

RNA was extracted from BC1 and BCBL1 cells treated with Mubritinib (15 nM), TPA (20 ng/ml)/NaB (2 mM), or DMSO control. Superscript IV RT (Invitrogen) was used to synthesize cDNA. qPCR was performed as above. Primers used for qPCR of KSHV genes include: ORF50 (5′-CAGCGTCCACTCCTGCAA-3′ and 5′-CCGGTGTTCTCTGCGACAA-3′); ORF45 (5′-CGTCCGGAGAGTTGGAACTG′ and 5′-GCGATCGTCGACCTGACAT-3′); PAN (5′-CGGTGTTTTGGCTGGGTTT′ and 5′-AAACCTTGCCGTCTGGTCACT-3′); LANA (5′-GAGTCTGGTGACGACTTGGAG3′ and 5′-AGGAAGGCCAGACTCTTCAAC-3′); ORF72 (5′-CATTGCCCGCCTCTATTATCA and 5′-ATGACGTTGGCAGGAACCA-3′); ORF71 (5′-TGCGACCTGCACGAAACA and 5′-GGAGGAGGGCAGGTTAACGT-3′). GUSB was used as a cellular control: GUSB (5′-CGCCCTGCCTATCTGTATTCand 5′-TCCCCACAGGGAGTGTGTAG-3′).

### Metabolomics

Cells were diluted with RPMI containing 2.5% heat-inactivated fetal bovine serum and penicillin and streptomycin as follows: BCBL1 (80,000 cell/mL), BJAB (24,000 cells/mL), BC1 (115,000 cells/mL), LCL352 (60,000 cells/mL). Then, the cells were seeded in a 15-cm dish and treated with 15 nM Mubritinib at a final DMSO concentration of 0.1%. As a control, growth media was treated with DMSO at a final concentration of 0.1%. Approximately 72 hours after treatment, the cells were collected by centrifugation, washed twice with ice cold PBS, and resuspended in extraction buffer containing 80% MeOH, 20% water, and heavy-labeled internal standards. Then, the samples were briefly vortexed and centrifuged at 13,000 rpm for 15 min and 4°C in a microcentrifuge. Supernatants were collected for analysis. The pellets were briefly dried and used to determine protein concentration for normalization. The metabolomics analysis was performed at The Wistar Institute Proteomics and Metabolomics Shared Resource on a Thermo Q-Exactive HF-X mass spectrometer. Each experiment was performed in triplicate.

### Mitochondrial function

We assessed the effect of Mubritinib (15 nM) treatment on mitochondrial function using the Seahorse XF Cell Mito Stress Kit assay (Agilent, UK). Briefly, BJAB, BC1, BCBL1, and LCL352 cells were treated overnight with Mubritnib (15 nM) or DMSO control. The day of the assay, 2 × 10^5^ live cells per well were plated in a 96 well plate in Seahorse medium (Agilent, UK) pre-coated with 22.4 μg/ml Cell-Tak™ (Corning, New York). The Seahorse assay was run per kit instructions. Concentrations of drugs to modulate respiration were optimized for each cell line based on empirical determination of optimal oxygen consumption rate (OCR) curves. For BJAB, BC1, and BCBL1 we used 2 μM oligomycin, 1 μM carbonyl cyanide-4 (trifluoromethoxy) phenylhydrazone (FCCP), 1 μM rotenone and antimycin A. In LCL352, the drug concentrations were reduced to used 1 μM oligomycin, 0.5 μM carbonyl cyanide-4 (trifluoromethoxy) phenylhydrazone (FCCP), and 0.51 μM rotenone and antimycin A.

## SUPPLEMENTARY MATERIALS



## Abbreviations

AAALAC: American Association for Accreditation of Laboratory Animal Care
ADP: adenosine diphosphate
AML: adult acute myeloid leukemia
AMP: adenosine monophosphate
ATP: adenosine triphosphate
ChIP: *chromatin* immunoprecipitation assay
CRISPR: clustered regularly interspaced short palindromic repeats
CPT: camptothecin
CYT: Cytarabine
DBD: deoxyribonucleic acid binding domain
DMSO: dimethyl sulfoxide
DNA: deoxyribonucleic acid
EBNA1: Epstein Barr virus Nuclear Antigen 1
EBV: Epstein Barr virus
ETC: electron transport chain
ErbB2: erb-b2 receptor tyrosine kinase 2
FCCP: phenylhydrazone
FITC: fluorescein isothiocyanate
HER2: human epidermal growth factor receptor 2
HHV-8: human herpesvirus 8
HRP: horseradish peroxidase
hTERT: human telomerase reverse transcriptase
IVIS CT: *in vivo* imaging system computerized tomography
KS: Kaposi’s sarcoma
KSHV: Kaposi’s sarcoma associated herpesvirus
KSHV TR: Kaposi’s sarcoma associated herpesvirus terminal repeats
LANA: latency-associated Nuclear Antigen
OXPHOS: oxidative phosphorylation
MCD: multicentric Castleman’s disease
MeOH: methanol
MLP: adenovirus major late *promoter;* MUB: Mubritinib
NAD: nicotinamide adenine dinucleotide
NADH: nicotinamide adenine dinucleotide
NRF2: nuclear factor erythroid2-related factor 2
OCR: oxygen consumption rate
ORF: open reading frame
PAN: polyadenylated nuclear RNA
PCR: polymerase chain reaction
PEL: primary effusion lymphoma
PI: propidium iodide
PO: by mouth, orally
QD: *quaque die,* once daily
qPCR: quantitative PCR
RAP: rapamycin
Rb: *retinoblastoma* tumor suppressor
RMCE-HILO: high-efficiency and low-background recombination- mediated cassette exchange technology
RPMI: Roswell Park Memorial Institute medium
ROCK: Rho-associated protein kinase
ROS: reactive oxygen species
RTK: receptor tyrosine kinase inhibitor
RTA: replication and transcription activator
