# Supplementary material for: Identification of Mubritinib (TAK 165) as an inhibitor of KSHV driven primary effusion lymphoma via disruption of mitochondrial OXPHOS metabolism
Source: Oncotarget. 2020 Nov 17;11(46):4224–42. doi: 10.18632/oncotarget.27815 (PMC7679036; doi:10.18632/oncotarget.27815)
Supplement: Supplementary file 1 [file oncotarget-11-4224-s001.pdf]

Identification of Mubritinib (TAK 165) as an inhibitor of KSHV driven primary effusion lymphoma via disruption of mitochondrial OXPHOS metabolism

SUPPLEMENTARY MATERIALS

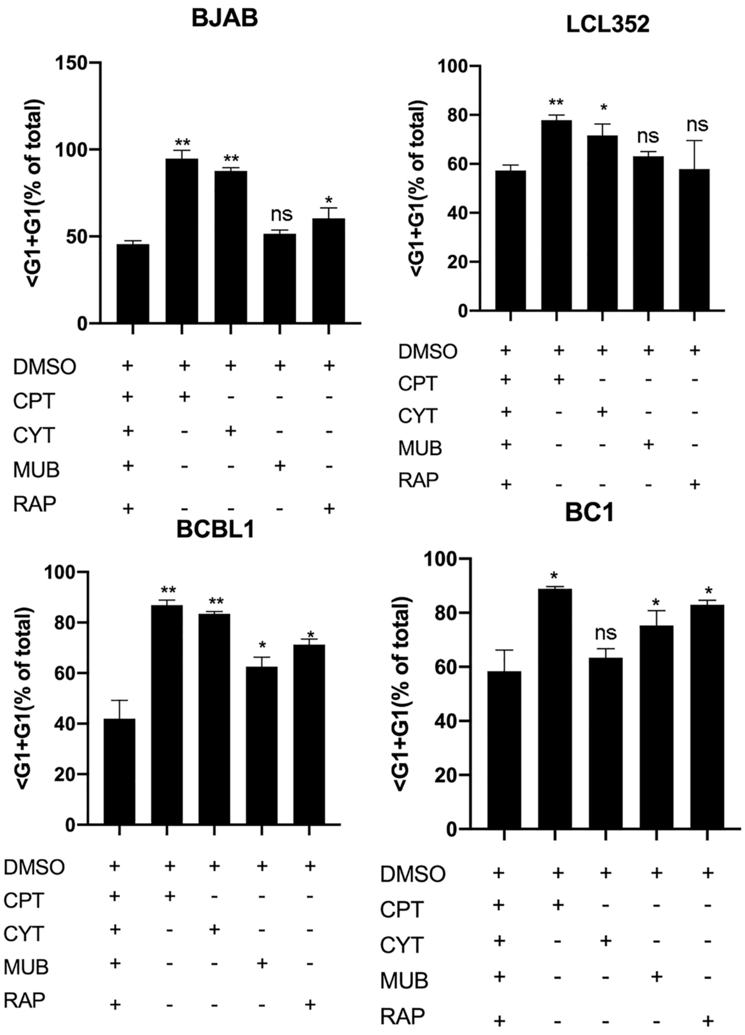

Supplementary Figure 1: Analysis of subG1 and G1 populations. SubG1 and G1 populations were quantified for cells treated in Figure 4. Mubritinib (MUB) to cytarabine (CYT) and rapamycin (RAP). DMSO and camptothecin (CPT) were used as controls. PEL cells (BC1 and BCBL1) treated with 15 nM Mubritinib show a selective increase in the sub G1 and G1 cells for BC1 and BCBL1 that is not observed for BJAB or LCL352 cells/($^{**}p < 0.001$, $^{*}p < 0.05$; Student's T Test).

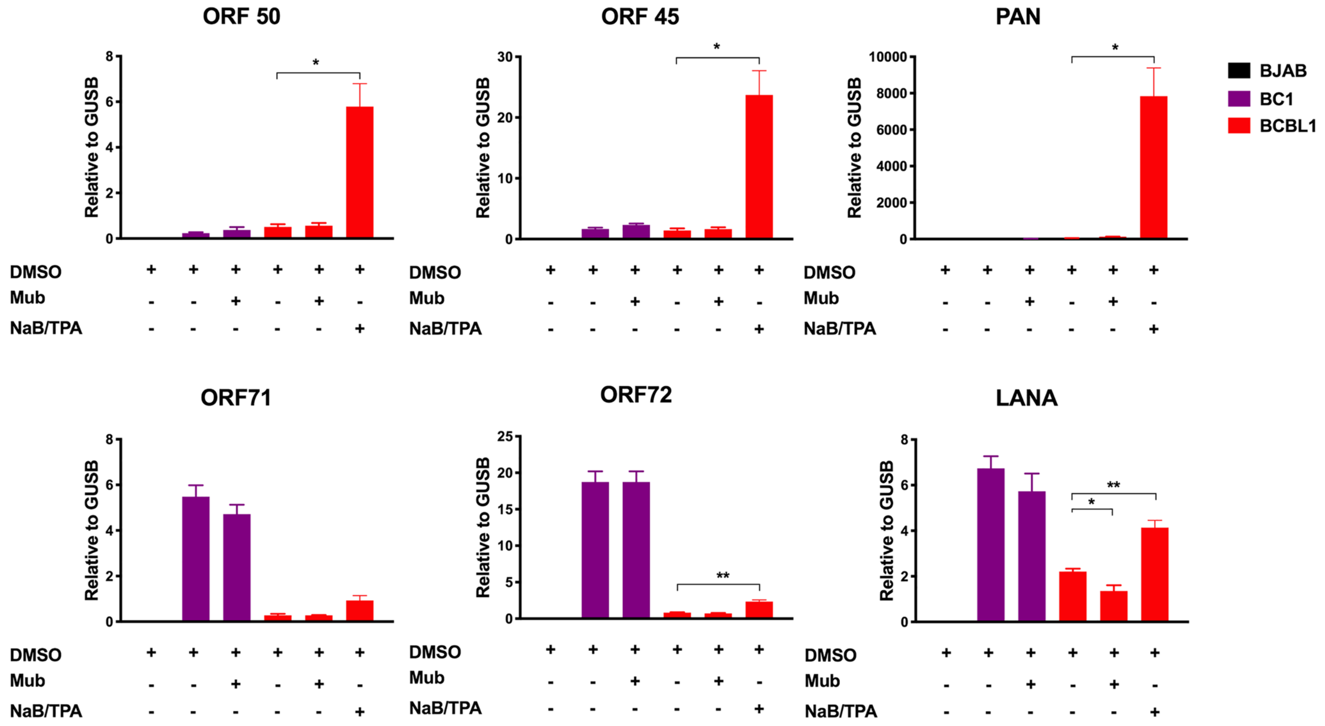

Supplementary Figure 2: Mubritinib does not activate KSHV lytic cycle gene expression. RT-PCR showing expression of KSHV ORF45, ORF 50, PAN, ORF71, ORF72, and LANA in BJAB, BC1, or BCBL1 cells. NaB/TPA was used for positive control of lytic induction. ($^{*}p < 0.05$, $^{**}p < 0.001$; *t*-test).

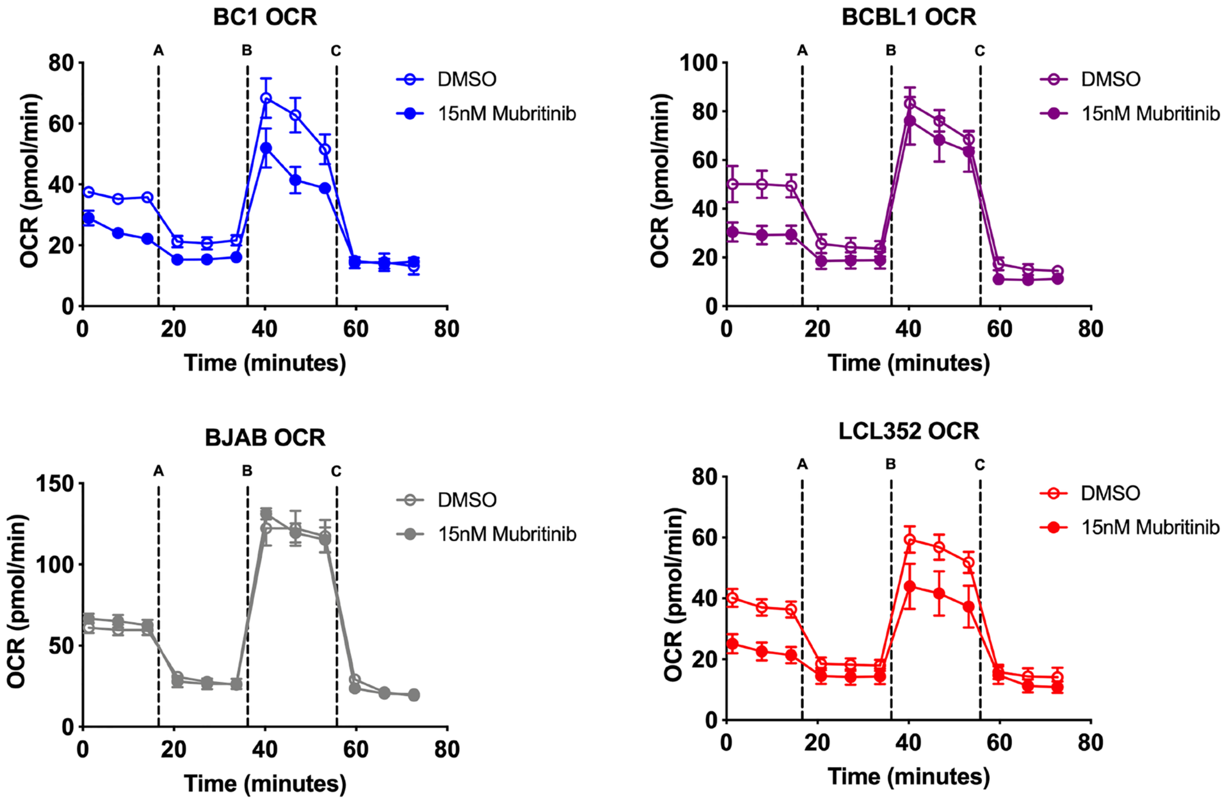

Supplementary Figure 3: Seahorse analysis of cells treated with Mubritinib. Oxygen consumption rates measured in BC1, BCBL1, BJAB, and LCL352 cells treated with (A) oligomycin (B) FCCP (C) rotenone and antimycin A.

Supplementary Table 1: GI_{50} Values for hits from the SelleckChem library

	BJAB	Ramos	BC3	BCBL1	BC1	LCL352
KSHV	−	−	+	+	+	−
EBV	−	−	−	−	+	+
Mubritinib	1.62 μM	0.16 μM	**13.45 nM**	**17.1 nM**	**7.49 nM**	0.38 μM
Linifanib	0.68 μM	0.42 μM	0.44 μM	0.54 μM	0.25 μM	0.44 μM
Regorafenib	0.43 μM	0.56 μM	**70.2 nM**	N.D.	**75.9 nM**	0.17 μM
Carmofur	11.85 μM	N.D.	0.74 μM	2.39 μM	1.38 μM	3.29 μM
Adrucil	6.18 μM	N.D.	0.23 μM	1.40 μM	0.49 μM	1.56 μM
Erlotinib HCl	N.D.	N.D.	0.63 μM	5.33 μM	1.16 μM	2.38 μM
Lenalidomide	N.D.	N.D.	**2.34 nM**	0.14 μM	0.39 μM	N.D.
OSI-420	7.10 μM	3.52 μM	**5.12 nM**	1.24 μM	0.13 μM	**57.45 nM**
Dopamine HCl	20.30 μM	20.40 μM	**3.39 nM**	14.47 μM	5.81 μM	6.15 μM
NSC-207895	9.74 μM	4.80 μM	N.D.	1.52 μM	0.81 μM	3.95 μM

Supplementary Table 2: GI_{50} Values for HER2/ErbB2 inhibitors and ETC inhibitors

	Ramos	BC3	BCBL1	BC1	LCL352
KSHV	−	+	+	+	−
EBV	−	−	−	+	+
Mubritinib	0.16 μM	**13.45 nM**	**17.1 nM**	**7.49 nM**	0.38 μM
Afatinib	0.18 μM	0.35 μM	0.12 μM	0.43 μM	0.78 μM
Dacomitinib	**81.2 nM**	**61.8 nM**	**0.13** μM	0.12 μM	0.16 μM
Emodin	27.60 μM	14.30 μM	17.60 μM	14.20 μM	25.70 μM
TAK-285	1.03 μM	1.12 μM	2.67 μM	1.23 μM	2.52 μM
Rotenone		**19.5 nM**	**16.1 nM**	**10.8 nM**	0.15 μM
Deguelin		1.16 μM	**44.2 nM**	**61.0 nM**	1.73 μM
